# Editorial: Revisiting Behavioral Variability: What It Reveals About Neural Circuit Structure and Function

**DOI:** 10.3389/fnbeh.2022.956388

**Published:** 2022-06-15

**Authors:** Kenta Asahina, Benjamin L. de Bivort, Ilona C. Grunwald Kadow, Nilay Yapici

**Affiliations:** ^1^Molecular Neurobiology Laboratory, The Salk Institute for Biological Studies, La Jolla, CA, United States; ^2^Center for Brain Science, Faculty of Arts and Sciences, Harvard University, Cambridge, MA, United States; ^3^Department of Organismic and Evolutionary Biology, Faculty of Arts and Sciences, Harvard University, Cambridge, MA, United States; ^4^Faculty of Medicine, Institute of Physiology II, University of Bonn, Bonn, Germany; ^5^Department of Neurobiology and Behavior, College of Arts and Sciences, Cornell University, Ithaca, NY, United States

**Keywords:** individuality, behavior, genetics, neuromodulation, behavioral variability, behavioral adaptation, neural circuit, internal state

Why is animal behavior variable? The main goal of this Research Topic is to showcase the latest research and perspectives that address this fundamental yet often overlooked question in behavioral neuroscience. Five original research articles and seven reviews by leading neuroscientists provide diverse insights on this question through various behavioral models.

Ethologists have long noted that animals and humans often respond differently to the same sensory stimuli. Although variability is common in nature, its study as an essential biological feature has faced friction in lingering ideas, such as small organisms being simple stimulus-response automata. Connectomes, complete maps of neural connectivity, are miraculous accomplishments, but their singular, structural nature can reinforce the feeling that nervous systems are non-varying. On the other hand, these data also revealed many previously unknown synaptic connections, suggesting more alternative routes between neurons and brain regions than necessary for simple stimulus-response routines. Indeed, numerous studies have demonstrated that even a well-defined neural circuit can produce a variety of behavioral outputs. Traditionally, different origins of behavioral variability have been studied in discrete frameworks (such as neural development, learning and memory, reproductive state, and so on). These distinctions do not necessarily reflect the differences in underlying mechanisms, which likely act in superposition in real organisms. To visualize the richness of mechanisms discussed in this Research Topic, we placed the areas covered by each article on a 2-dimensional map. One axis represents the timescale of behavioral variability, and the other axis represents its mechanistic levels (Figure 1).

**Figure 1:**
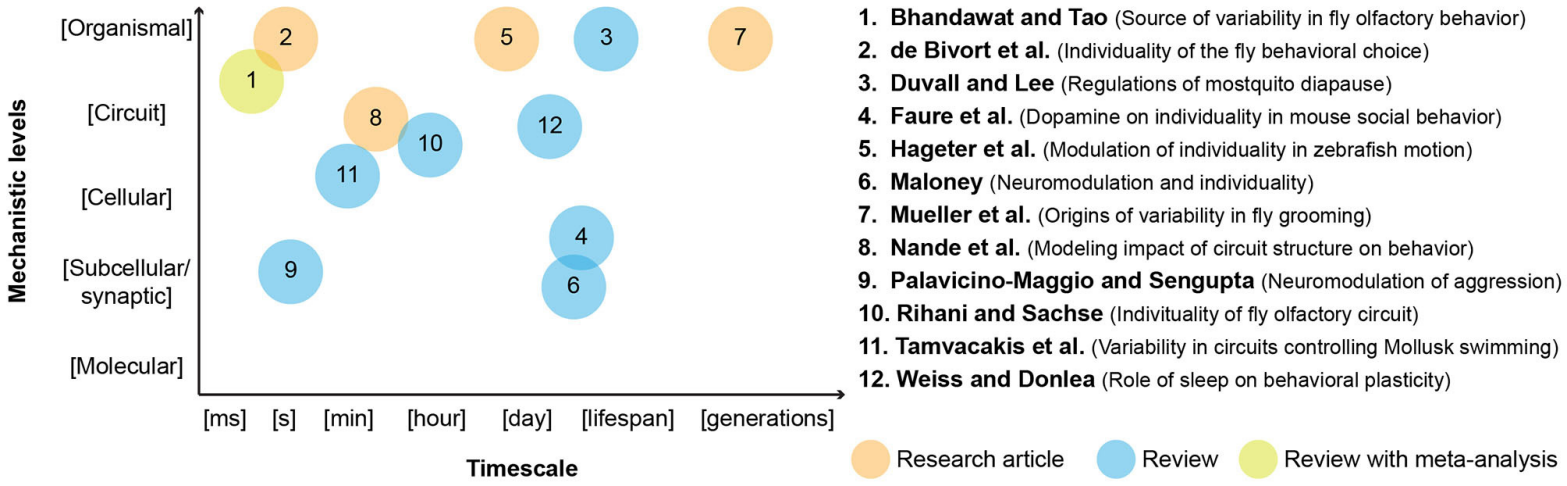
Mapping the scope of articles in this Research Topic. Numbers are given according to the alphabetical order of the first authors.

The term “variability” often refers to a within-group difference in observable behavioral outputs that cannot be explained by the factor of interest, e.g., a stimulus or genetic variations within the group. For instance, Darwin was unaware that finches in the Galapagos Islands consisted of multiple species until his colleague ornithologist John Gould pointed that out. In this case, what was initially perceived as anatomical variability within a group turned out to be species-specific characteristics. Knowledge on taxonomy coupled with rigorous quantification of behavior helps distinguish intra- and inter-specific variabilities, as shown by Mueller et al. While it is clear that inter-specific variability is caused by heritable genetic differences between species (though any co-varying environmental effects may also contribute), within-species variabilities might also arise from non-heritable causes such as noise in gene expression due to environmental factors. Both heritable and non-heritable variations affect behavior through multiple cellular and physiological mechanisms, including varying circuit connectivity. Using olfactory-guided behavior of the common fruit fly *Drosophila melanogaster*, Tao and Bhandawat discuss potential genetic contributions for behavioral variability, while Rihani and Sachse illustrate variabilities in neuroanatomy and physiological properties of neural circuits that can be the source of individual behavioral differences. In parallel, Tamvacakis et al. discuss the impact of variability in circuit wiring and gene expression patterns in key neurons driving flexibility in mollusk swimming behavior.

Besides wiring variation, an alternative source of variability is multiple, discrete developmental programs within a species. Lee and Duvall consider egg diapause, an alternative state of arrested development under harsh environmental conditions, in the mosquito *Aedes albopictus*. This is an intriguing example of how external factors drive alternative reproductive strategies within a genetically homogeneous population. Similarly, Hageter et al. demonstrate that temperature fluctuations during Zebrafish development affect specific aspects of turning behavior. Another example is the effect of social experiences, which can profoundly impact animal behavior. Faure et al. discuss how complex social interactions in rodents can reinforce individual differences with significant fitness consequences. As discussed in the above three papers, specific genes likely play an essential role in converting experience during different development timescales into behavioral adaptations. Recent advances in sequencing technology can illuminate key genetic networks that are important for generating behavioral variability in response to changes in environmental conditions.

Genetic, environmental, and stochastic factors underlie stable behavioral idiosyncrasies, but that is not the only source of variability. The same animal often behaves differently when tested at different times, suggesting that parallel factors cause intra-individual fluctuations in behavior. The so-called “internal state” is often used without a clear scientific definition, but several types of “internal states” have been well-studied across species; among them is the general arousal state. Weiss and Donlea discuss how sleep (or the lack of it) can impact the neural functions of developing and mature brains, along with the behavioral consequences of sleep disruption. Arousal levels can be controlled in a behavior-specific manner as well. Palavicino-Maggio and Sengupta describe neurogenetic factors—namely neuromodulators—affecting aggression in *Drosophila melanogaster*. Across animal species, neuromodulation is a key to generating behavioral variability within and among individuals. Underscoring its importance, many articles in this Research Topic touch upon neuromodulation: Faure et al., Tamvacakis et al., Tao and Bhandawat, de Bivort et al., and Rihani and Sachse all discuss the significance of neuromodulation in the context of their behavioral paradigms. A review by Maloney argues that neuromodulation can drive behavioral variability by diversifying the dynamics of a circuit that controls a given behavior. Since many neuromodulators have similar behavioral effects across species, the cellular mechanisms of neuromodulation are critical to understanding how the nervous systems with (largely) identical connectivity can generate variable behavioral outcomes within and across individuals.

While distinguishing inter- and intra-individual variability seems straightforward in concept, a large amount of data and repeated measures from the same individual are often necessary to distinguish these two variabilities (see also Tamvacakis et al.). “Big data” of behavior have become amenable for analysis relatively recently thanks to newly developed computational and experimental toolkits. de Bivort et al., Mueller et al., and Hageter et al. showcase the power of behavioral data collected from a large number of animals when isolating biases characteristic of each animal—or individuality. In all three articles, it is noteworthy that individual behavioral biases are represented as probabilities of exhibiting particular choices rather than the simple presence or absence of a given behavior. Thus, individuality may be expressed as differences in the sequencing or abundance of behaviors rather than their kinematics. Through the meta-analysis of published data, Tao and Bhandawat found that stochastic choice likely generates larger inter-individual variability in olfactory-guided behavior than genetic or neuromodulatory differences. How stochasticity arises in the nervous system remains an important question in neuroscience. Nande et al. demonstrated through modeling that behavior-specific modular organization of the nervous system makes the behavioral output more robust against perturbation while imparting long-term internal-state-like dynamics. In other words, the difference between what is regarded as a “stereotypical behavior” and a “variable behavior” may reflect differences in the way the neural circuits that control the given behaviors are structured.

The diverse aspects of behavioral variability covered in this Research Topic compel us to ask whether these phenomena can be explained under a single framework. Even a “simple” nervous system turns out to be complex enough to generate behavioral variability. Despite large-scale neural recordings and flourishing “omics” data from molecules to behavior, the level of our current understanding of gene expression regulation, synaptic plasticity, neuromodulation, and circuit development and reorganization still seems insufficient to create cell and circuit models that provide quantitative hypotheses to account for behavioral variability. Rigorous behavioral analysis will also be critical but almost certainly insufficient. Scientists and editors alike love “clean” behavioral data with small error bars that fit together into tidy neurobiological narratives. But the exclusive pursuit of such results limits progress in identifying the origins of behavioral variability, which is so salient to every scientist who performs a behavioral experiment. We hope this Research Topic advances variability discourse in the behavioral neuroscience community and brings us a few steps closer to a mechanistic understanding of the neural functions that generate behavioral variability.

## Author Contributions

KA wrote the initial draft, which was substantially edited by all four co-authors. All the co-authors approved the final manuscript for publication. All authors listed have made a substantial, direct, and intellectual contribution to the work and approved it for publication.

## Funding

KA would like to acknowledge support from NIH NIGMS R35GM119844.

## Conflict of Interest

The authors declare that the research was conducted in the absence of any commercial or financial relationships that could be construed as a potential conflict of interest.

## Publisher's Note

All claims expressed in this article are solely those of the authors and do not necessarily represent those of their affiliated organizations, or those of the publisher, the editors and the reviewers. Any product that may be evaluated in this article, or claim that may be made by its manufacturer, is not guaranteed or endorsed by the publisher.

